# Association between patent foramen ovale and anxiety: a prospective study with deep learning-assisted analysis of contrast echocardiography

**DOI:** 10.3389/fpsyt.2026.1877855

**Published:** 2026-09-30

**Authors:** Minqi Liao, Changji Yang, Chengyu Hong, Xiaoyu Liu, Zheng Li, Shumin Ouyang, Xuande Zhang, Hanyu Weng, Ziying Cao, Zhihao Wu, Jiahua Ye, Zhichao Huang, Yanhua Yang, Kangmin Wang, Patience Ratidzo Ngombengombe, Jiongbin Lu, Zekui Wu, Zhanyang Wei, Mark T. K. Tam, Jianping Zhou, Xin Huang, Suxia Guo

**Affiliations:** 1 Department of Cardiology, Dongguan People’s Hospital (The Tenth Affiliated Hospital of Southern Medical University), Dongguan, China; 2 School of Electronic Information and Artificial Intelligence, Shaanxi University of Science & Technology, Xi’an, China; 3 Department of Neurology, Dongguan People’s Hospital (The Tenth Affiliated Hospital of Southern Medical University), Dongguan, China; 4 Department of Cardiovascular and Neurology, Dongguan Xiegang Hospital, Dongguan, China; 5 Department of Cardiology, Puji Campus of Dongguan People’s Hospital, Dongguan, China; 6 Division of Cardiology, Department of Medicine and Therapeutics, Faculty of Medicine, The Chinese University of Hong Kong, Hong Kong, Hong Kong SAR, China; 7 Department of Thoracic Surgery, Dongguan People’s Hospital (The Tenth Affiliated Hospital of Southern Medical University), Dongguan, China; 8 Faculty of Electrical Engineering and Computer Science, Ningbo University, Ningbo, China

**Keywords:** anxiety, contrast echocardiography, deep learning, heart-brain axis, patent foramen ovale

## Abstract

**Purpose:**

To prospectively investigate the association between patent foramen ovale (PFO), detected via agitated saline contrast echocardiography (ASCE), and anxiety using a deep learning approach.

**Materials and methods:**

This prospective study consecutively enrolled 161 participants between July 15, 2025 and October 31, 2025. Participants were stratified into ASCE-negative (n=58) and ASCE-positive (n=103) groups based on the examination results. All participants underwent ASCE and anxiety assessment using the Self-Rating Anxiety Scale (SAS). An artificial intelligence model was developed using a 10-fold cross-validation approach on the 103 ASCE-positive videos to predict anxiety scores. The primary endpoint was the association between PFO and anxiety, while the deep learning model was evaluated as a secondary/exploratory endpoint. Statistical analyses, including logistic regression, were performed with statistical significance set at p<0.05.

**Results:**

Compared to the ASCE-negative group, the ASCE-positive group exhibited significantly higher anxiety levels (SAS score: 45.0 vs. 38.0, p<0.001) and a higher prevalence of anxiety (32.0% vs. 5.2%, p<0.001). Logistic regression identified a strong association between ASCE-positive status and anxiety (adjusted odds ratio=8.64, p<0.001), with no clear dose-dependent relationship across shunt grades. The imaging-only deep learning model achieved a mean absolute error (MAE) of 5.76 ± 0.12 and an R² of 0.46 ± 0.03 across cross-validation folds. After incorporating clinical and sociodemographic confounders, the model’s performance improved to an MAE of 5.15 ± 0.10 and an R² of 0.59 ± 0.02.

**Conclusion:**

This prospective study confirms a significant association between PFO and anxiety and demonstrates the feasibility of using ASCE-based deep learning models to predict anxiety risk, although clinical application requires external validation.

## 1 Introduction

The clinical overlap between cardiovascular and neuropsychiatric disorders, particularly in patients with patent foramen ovale (PFO), represents a significant but underexplored challenge in the general hospital setting. PFO, a persistent fetal interatrial communication, is present in approximately 25% of the adult population. While its role in cryptogenic stroke and migraine is well-established (1–3), a growing number of clinical observations suggest a potential, yet poorly defined, link between PFO and mood disorders, especially anxiety (4–6). While the mechanisms remain elusive, preliminary data imply that PFO-related hemodynamic or microembolic phenomena may perturb cerebral circuits involved in emotional regulation (7, 8).Crucially, for clinicians in cardiology or neurology departments, anxiety in these patients often remains unrecognized, complicating management and potentially exacerbating patient-reported symptoms. A preliminary report (9) that PFO closure can ameliorate anxiety symptoms further underscores the pressing need to systematically investigate this potential heart–mind connection.

Recent studies have begun to quantify this association more systematically. Skoczek et al. (5) reported psychological distress in PFO patients, while Barbosa et al. (6) and Zhai et al. (4) presented case reports highlighting psychiatric symptoms masking or preceding PFO-related presentations. More recently, Zhai et al. (10) conducted the first preliminary cohort study specifically examining anxiety and PFO in a Chinese population, reporting elevated anxiety scores and a significant correlation with shunt grade; however, their sample size was smaller (n=89) and they did not employ artificial intelligence-based imaging analysis. Similarly, Sun et al. (9) investigated clinical characteristics of migraine with PFO and found a high prevalence of anxiety symptoms in that subgroup, though they did not isolate the PFO-anxiety link independent of migraine. Collectively, these prior studies were largely observational, case-based, or lacked systematic quantification using validated scales, and none utilized AI-based imaging analysis. Our study provides several advances: (i) prospective design with validated SAS questionnaire; (ii) blinded assessment to minimize bias; (iii) stratification by ASCE grade to examine dose-response relationships; and (iv) novel application of deep learning to ASCE videos for anxiety prediction. To our knowledge, this is the first study to combine systematic anxiety assessment with AI-based analysis of contrast echocardiography in PFO patients, providing a methodological innovation that distinguishes it from previous work in this field.

The primary objective of this study was, therefore, to test the hypothesis that PFO, detected via agitated saline contrast echocardiography (ASCE), is independently associated with anxiety. We aimed to quantify this association beyond the effects of traditional sociodemographic and clinical risk factors. Given that conventional ASCE interpretation is subjective and qualitative, we further deployed a deep learning framework — leveraging a pre-trained video encoder called EchoPrime (11) — as an explorative tool to extract imaging biomarkers from ASCE videos and predict anxiety scores. This approach allows us to move beyond simple bubble-counting and explore whether subtler, machine-identified hemodynamic features correlate with emotional dysregulation. Through this prospective design, we sought to provide clinicians with a novel, imaging-based marker for identifying anxiety risk in patients undergoing routine contrast echocardiography.

## 2 Methods

### 2.1 Study design and participants

This prospective cohort study (Chinese Clinical Trial Registry: ChiCTR2500105966) consecutively included patients who visited the cardiology/neurology departments of Dongguan People’s Hospital, Puji Campus of Dongguan People’s Hospital, Dongguan Xiegang Hospital (China), between July 15, 2025 and October 31, 2025. Clinicians suspected abnormal cardiac structure and evaluated internal shunting, abnormal embolism, unexplained stroke, transient ischemic attack, migraine, etc. The inclusion criteria were: 1) patients who were clinically referred for agitated saline contrast echocardiography based on suspected cardiac structural abnormalities or right-to-left shunting; 2) age 18–65 years; 3) completion of the SAS questionnaire; and 4) voluntary provision of written informed consent. The exclusion criteria included: 1) previous diagnosis of anxiety/depression; 2) current treatment with anti-anxiety/depression medication; 3) presence of other serious mental and psychological disorders (e.g., schizophrenia, insomnia, personality disorders, etc.) or the use of antipsychotic drugs; 4) low quality of imaging; and 5) incomplete clinical data. All enrolled patients were stratified into ASCE-negative and ASCE-positive groups according to the examination results.

### 2.2 Data collection

The investigation involved the collection of clinical data on factors that affect anxiety status (e.g., age, sex, SAS, stirred saline contrast echocardiography imaging, marital status, stable income, migraine, family history of mental illness, chronic medical conditions, use of hormone drugs, beta blockers, education level, smoking, alcohol consumption, and coffee consumption).

### 2.3 Blinding

Given the subjective nature of anxiety assessment, blinding procedures were implemented to minimize bias. Patients were not informed of their ASCE results at the time of completing the anxiety self-assessment. Similarly, the echocardiography interpreters were blinded to the patients’ anxiety scores during image analysis.

### 2.4 Deep learning model development

The deep learning model was developed using ASCE-positive videos. We adopted the EchoPrime framework (12), which was a video-based multiview vision-language foundation model pre-trained using the Contrastive Language-Image Pre-training method on a dataset containing over 12 million video-report pairs. In its architecture, EchoPrime employed MVIT v2 (13) as the video encoder and BioMedBERT(https://github.com/BioMedBERT/biomedbert) as the text encoder.

Compared with other research fields, obtaining right heart echocardiographic data and anxiety diagnosis results from patients with PFO was more difficult, and there were higher professional requirements for data annotators. This made obtaining a large amount of high-quality annotated data a major challenge in the model development process. Given that EchoPrime has completed pre-training with large-scale echocardiography datasets and demonstrated excellent performance in multiple tasks, we utilized it as a model encoder to introduce the feature extraction ability learned by EchoPrime in large-scale echocardiography data training.

The model architecture of this study consisted of two core components: 1) a video encoder for extracting spatiotemporal features of echocardiography; and 2) a decoder used for anxiety prediction. Specifically, EchoPrime pre-trained MVIT V2 was used as the video encoder to extract spatiotemporal features of echocardiography imaging data. The encoder adopted the classic configuration of MVIT V2 and included 16 Multiscale Vision Block modules. Only the output dimension of multilayer perceptron in the last Multiscale Vision Block module was adjusted from 768 dimensions to 512 dimensions, while the other parameters remained unchanged. The decoder adopted a multilayer perceptron architecture and predicted the patient’s anxiety level based on the features extracted by the encoder (**Figure 1**).

**Figure 1:**
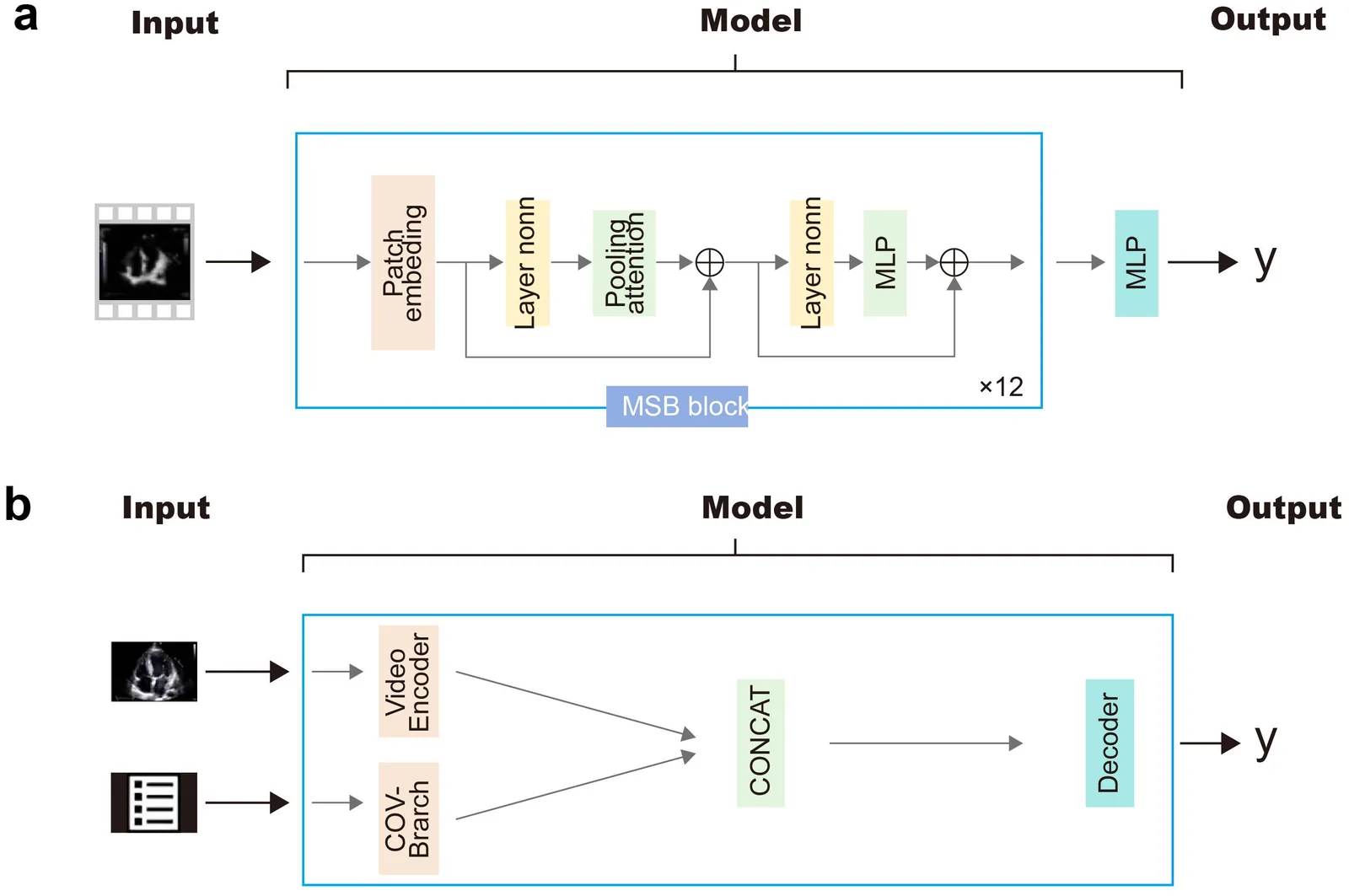
**(a)** Model network structure diagram **(b)** Network structure diagram of dual-path fusion model.

Building upon imaging-based anxiety assessment, we developed a multimodal fusion framework that integrates an imaging stream with a clinical covariate branch. The imaging branch utilized spatiotemporal embeddings extracted from agitated saline contrast echocardiography (ASCE) video streams via an EchoPrime encoder. Concurrently, the covariate branch processed a suite of structured clinical variables, encompassing demographics (age, sex, education, marital status, and stable income), medical history (migraine, chronic diseases, and family history of psychiatric disorders), and lifestyle or pharmacological factors (smoking, alcohol and coffee consumption, and the use of hormonal or β-receptor blockers). These dual-stream features were fused at the representation level and subsequently mapped to continuous Self-Rating Anxiety Scale (SAS) scores through a regression head. To quantify the incremental predictive value of clinical information, we evaluated the performance gain of this integrated multimodal model over an imaging-only baseline (**Figure 1**).

In this study, 16 frames of images from the original echocardiogram data with a step size of 2 were randomly selected, and a bicubic algorithm was used to scale the spatial resolution of each frame to 224 × 224 × 3. From the original echocardiographic frame sequence, 16 frames were sampled with a fixed interval and random start: if the number of original frames (N) was ≥ 31 (since sampling 16 frames at a step size of 2 requires covering the range from the start frame to, the optional range for is), one was randomly selected from this range as the starting frame, and 16 consecutive frames were selected starting from at a step size of 2; if the length of the original frame sequence (N) was less than 31, the first frame was used as the starting frame, all available frames were sampled at a step size of 2, and pixel-level zero-padding was added to the end of the sequence until the total number of frames reached 16.The sampled video was standardized using mean and standard deviation, and the standardized data were input into the video encoder for feature extraction. The features extracted by the encoder were directly input into the decoder, which predicts the patient’s anxiety level based on the features.

The choice of 16 frames was constrained by the architectural requirements of the pre-trained EchoPrime foundation model (MViT V2), which was originally trained with a fixed input sequence length of 16 frames. To leverage the pre-trained weights (which were kept frozen during training), we sampled exactly 16 frames from each ASCE video. The step size of 2 was selected to balance temporal coverage and computational efficiency.

The model training adopted AdamW optimizer (β_1_ = 0.9, β_2_ = 0.99), combined with cosine learning rate scheduler, with an initial learning rate of 1×10^-3^ and a minimum learning rate of 1×10^-6^. The batch size was set to 64 and training for 50 epochs was conducted using a NVIDIA A100-40G graphics card. During the training process, the encoder parameters were fixed and only the decoder parameters were updated. Therefore, the feature extraction ability of the model was completely determined by the EchoPrime pre-training parameters.

To obtain robust and unbiased performance estimates, we employed 10-fold cross-validation on the 103 ASCE-positive videos. The dataset was randomly partitioned into 10 equal-sized folds; in each iteration, 9 folds were used for training and the remaining 1 fold for validation, with the process repeated 10 times. The reported performance metrics (MAE and R²) were presented as the mean ± standard deviation across all 10 folds. This approach provided a more reliable estimate of model generalizability than a single train–validation split and mitigated the risk of overfitting.

### 2.5 Statistical information

The primary endpoint of this study was to evaluate the independent association between PFO and anxiety using logistic regression. Inter-observer reliability for ASCE shunt grading was assessed by two independent echocardiographers on a random sample of 40 videos using Cohen’s kappa coefficient. The deep learning model development was designated as a secondary endpoint to explore the feasibility of imaging-based anxiety prediction. The full cohort was used for clinical characteristic comparisons and logistic regression analyses. The deep learning model was developed on ASCE-positive videos. Statistical analysis was conducted using SPSS 27.0 software (IBM Corp., Armonk, NY, USA). The comparison between count data groups was conducted using the chi-squared test. The metric data of normal distribution are represented by (x ± s), and analysis of variance was used for inter-group comparison. Non-normally distributed quantitative data are represented by median and interquartile range, and inter-group comparisons were conducted using the *t*-test or rank-sum test.

## 3 Results

A total of 181 patients were assessed for eligibility, of whom 20 were excluded: 13 due to incomplete clinical data (primarily missing SAS scores or key demographic variables) and 7 due to low imaging quality. No imputation was performed for missing data; only complete cases were included in the final analysis. The final cohort comprised 161 participants, who were stratified into ASCE-negative (n=58, 36.0%) and ASCE-positive (n=103, 64.0%) groups. Inter-observer agreement for ASCE shunt grading was excellent, with a Cohen’s kappa of 0.87 (95% CI: 0.79–0.95). The 103 ASCE-positive videos were subsequently used for deep learning model development, whereas the full cohort was used for clinical comparisons and logistic regression analyses. Key demographic and clinical characteristics are presented in **Table 1**. No significant differences were observed in age (mean: 41.7 ± 11.4 years; 43.9 ± 11.5 vs. 40.4 ± 11.2, respectively, p=0.45), sex distribution (male: 43.5%; 48.3% vs. 40.8%, respectively, p=0.06), or marital status (married: 77.6%; 86.2% vs. 72.8%, respectively, p=0.08). The groups were characterized by comparable education levels (p=0.18), with undergraduate degree being the most common (46.0% overall). No significant group differences were found in migraine prevalence (22.9% overall), family history of mental illness (34.2%), or chronic diseases (34.2%). Notably, coffee consumption patterns differed, with the positive group showing more frequent consumption than the negative group (27.2% vs. 12.1%, respectively, p=0.046). Smoking and drinking habits were comparable between groups (p>0.10). Compared with the ASCE-negative group, the ASCE-positive group demonstrated significantly higher Self-Rating Anxiety Scale (SAS) scores (median [interquartile range]: 38.0 [33.3–42.0] vs. 45.0 [39.5–53.0], respectively, p<0.001) and anxiety prevalence (5.2% vs. 32.0%, respectively, p<0.001). The severity of anxiety differed markedly (p=0.001), with 19.4% mild, 8.7% moderate, and 3.8% severe cases in the positive group versus 3.4% mild and 1.7% moderate cases in the negative group.

**Table 1:** Characteristics of the study participants by whether the ASCE imaging is positive.

ASCE	Total (n=161)	Negative (n=58)	Positive (n=103)	P-value
Age, years	41.67 (11.39)	43.90 (11.49)	40.40 (11.19)	0.450
Male, n (%)	70 (43.5)	28 (48.3)	42 (40.8)	0.060
SAS score	42.00 (36.00-49.00)	38.00 (33.25-42.00)	45.00 (39.50-53.00)	<0.001
Anxiety, n (%)	36 (22.4)	3 (5.2)	33 (32.0)	<0.001
Anxiety Level, n (%)				0.001
Mild	22 (13.7)	2 (3.5)	20 (19.4)	
Moderate	10 (6.2)	1 (1.7)	9 (8.7)	
Severe	4 (2.4)	0 (0.0)	4 (3.8)	
Marriage, n (%)	125 (77.6)	50 (86.21)	75 (72.8)	0.080
Stable income, n (%)	136 (84.5)	52 (89.7)	84 (81.6)	0.260
Migraine, n (%)	37 (23.0)	10 (17.2)	27 (26.2)	0.270
Family history of mental illness, n (%)	55 (34.2)	19 (32.8)	36 (35.0)	0.910
Chronic disease, n (%)	55 (34.2)	23 (39.7)	32 (31.1)	0.350
Hormone drugs, n (%)	38 (23.6)	14 (24.1)	24 (23.3)	1.000
β receptor blocker, n (%)	55 (34.2)	23 (39.7)	32 (31.1)	0.350
Education, n (%)				0.180
Primary school	18 (11.2)	10 (17.2)	8 (7.8)	
Middle school	42 (26.1)	17 (29.3)	25 (24.3)	
Undergraduate	74 (46.0)	24 (41.4)	50 (48.5)	
Graduate student	27 (16.8)	7 (12.1)	20 (19.4)	
Smoking, n (%)				0.100
Never	85 (52.8)	36 (62.1)	49 (47.6)	
Occasionally	67 (41.6)	21 (36.2)	46 (44.7)	
Frequently	9 (5.6)	1 (1.7)	8 (7.8)	
Drinking, n (%)				0.920
Never	57 (35.4)	22 (37.9)	35 (34.0)	
Occasionally	72 (44.7)	26 (44.8)	46 (44.7)	
Frequently	22 (13.7)	7 (12.1)	15 (14.6)	
Everyday	10 (6.2)	3 (5.2)	7 (6.8)	
Coffee, n (%)				0.046
Never	4 (2.5)	0 (0.0)	4 (3.9)	
Occasionally	104 (64.6)	44 (75.9)	60 (58.3)	
Frequently	35 (21.7)	7 (12.1)	28 (27.2)	
Everyday	18 (11.2)	7 (12.1)	11 (10.7)	

The correlation heatmap revealed a correlation between PFO and anxiety symptoms, while showing minimal associations with other psychiatric risk factors (**Figure 2**). Specific values can be found in the **Supplementary Materials**. This specificity strengthens the hypothesis of a potential direct pathophysiological link, although the moderate effect size (r=0.31, p<0.001) suggests the involvement of additional moderating factors.

**Figure 2:**
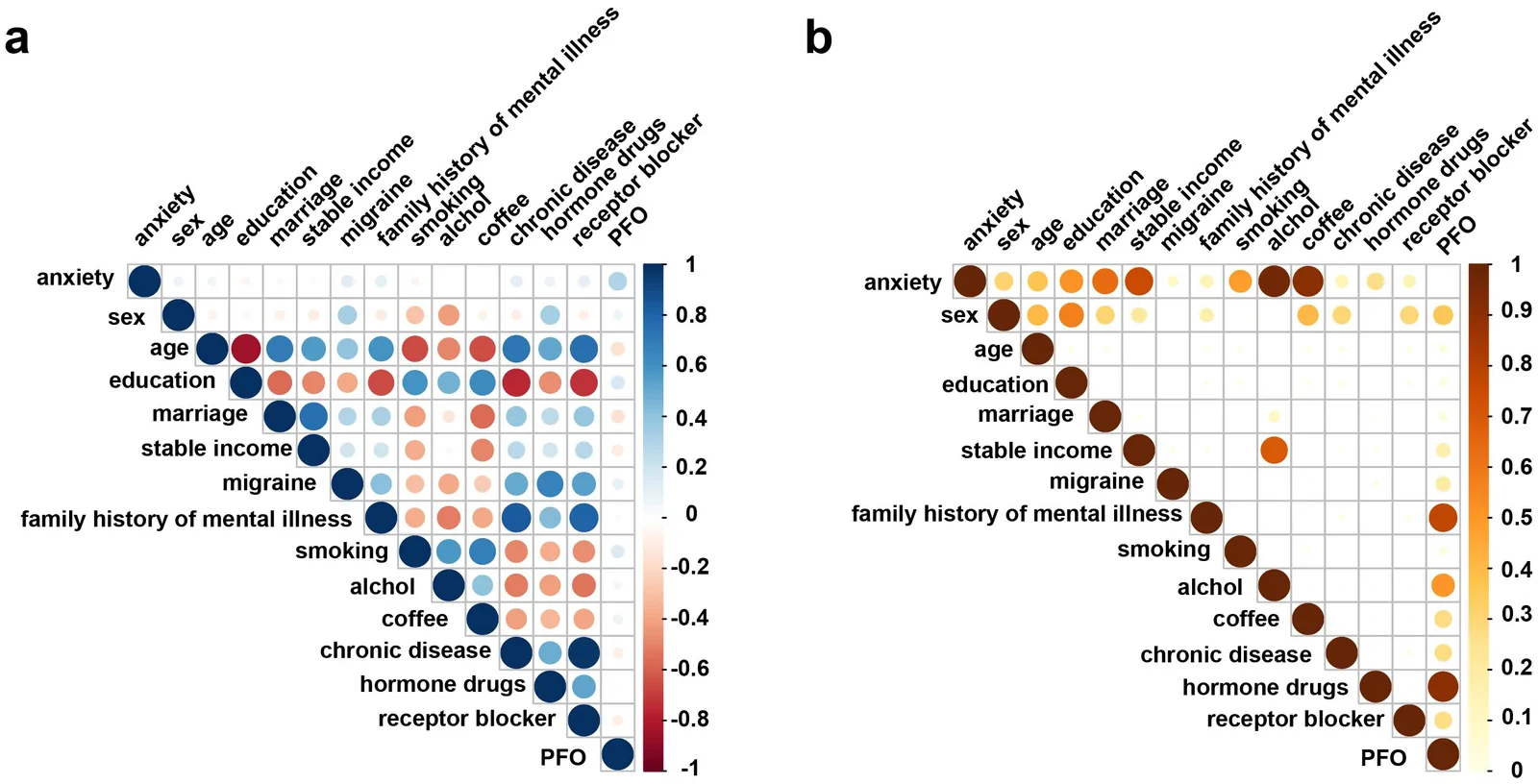
Correlation matrix of clinical variables. Panel **(a)** displays correlation coefficients (r), with red indicating positive correlation and blue indicating negative correlation; color intensity reflects the magnitude of correlation. Panel **(b)** displays the corresponding p-values for each correlation, with the color scale ranging from 0 to 1, where deeper red indicates smaller p-values (stronger statistical significance). For the full correlation matrix with exact r and p values, please refer to **Supplementary Table 1**.

The logistic regression analyses demonstrated a robust association between ASCE-positive status and anxiety (**Table 2**). In the crude model, ASCE-positive participants had 9.76-fold higher odds of anxiety compared with ASCE-negative participants (odds ratio [OR]=9.76, 95% confidence interval: 2.78–34.26, p<0.001). All ASCE-positive grades were associated with significantly elevated anxiety risk compared with grade 0 (all p<0.01), with adjusted ORs ranging from 7.76 to 9.17 (**Table 2**).

**Table 2:** Association between ASCE status/grading and anxiety: unadjusted and adjusted logistic regression models.

ASCE category	Crude model OR (95% CI)	P-value	Model I OR (95% CI)	P-value	Model II OR (95% CI)	P-value
Binary ASCE						
Negative (Ref.)	1.00	–	1.00	–	1.00	–
Positive	9.76 (2.78, 34.26)	<0.001	9.64 (2.55, 36.49)	0.001	8.64 (2.52, 29.67)	0.001
ASCE Grading						
Grade 0 (Ref.,n=58)	1.00	–	1.00	–	1.00	–
Grade 1 (n=42)	11.32 (2.88, 44.54)	0.001	10.14 (2.26, 45.49)	0.002	9.17 (2.43, 34.57)	0.001
Grade 2 (n=24)	9.95 (2.31, 42.79)	0.002	9.73 (2.10, 45.06)	0.004	9.17 (2.17, 38.65)	0.003
Grade 3 (n=37)	8.34 (2.10, 33.05)	0.003	9.10 (2.06, 40.19)	0.004	7.76 (1.99, 30.19)	0.003

ASCE shunt grades were defined based on bubble count in the left heart chambers: Grade 0 (no bubbles), Grade 1 (1–10 bubbles), Grade 2 (11–30 bubbles), Grade 3 (>30 bubbles or curtain pattern). Inter-observer agreement was assessed by two independent echocardiographers on a random sample of 40 videos: Cohen’s κ = 0.87 (95% CI: 0.79–0.95).

Model I (adjusted for age, sex).

Model II (further adjusted for marital status, stable income, migraine, family history of mental illness, chronic medical history, use of hormone drugs, beta blockers, education level, smoking, alcohol consumption, and coffee consumption).

The 103 ASCE-positive videos were analyzed using a 10-fold cross-validation strategy to obtain robust and unbiased performance estimates. The model’s performance across the 10 validation folds is shown in **Figure 3**. The imaging-only model (**Figure 3a**) achieved a mean absolute error (MAE) of 5.76 ± 0.12 and an R² of 0.46 ± 0.03 across the 10 folds. After incorporating age and sex as covariates (**Figure 3b**), the performance improved to an MAE of 5.48 ± 0.11 and an R² of 0.52 ± 0.03. Following further adjustment for all clinical and sociodemographic confounders—including marital status, stable income, migraine, family history, smoking, alcohol consumption, coffee intake, chronic diseases, use of hormonal medications, and beta-blockers—the model achieved its best performance (**Figure 3c**), with an MAE of 5.15 ± 0.10 and an R² of 0.59 ± 0.02. The narrow standard deviations across folds indicate stable, non-overfitted performance. These results demonstrate that integrating imaging data with clinical covariates progressively enhances the predictive accuracy for anxiety scores in patients with PFO.

**Figure 3:**
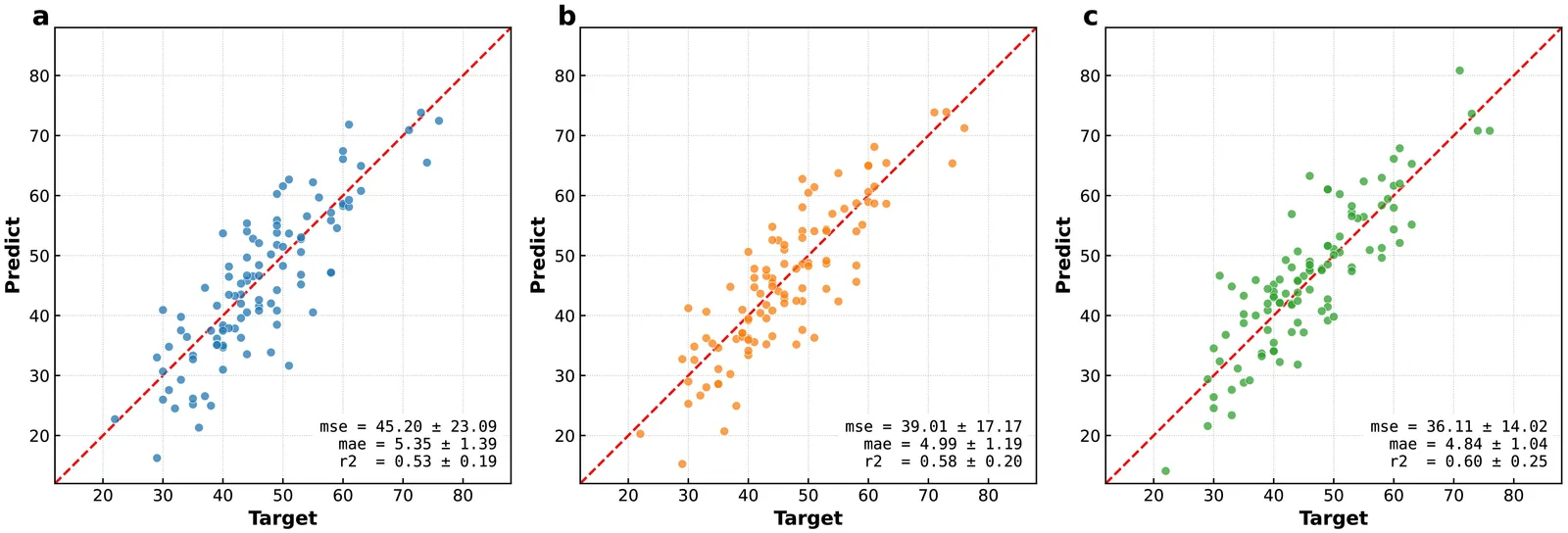
Predictive performance on the internal validation set using 10-fold cross-validation. **(a)** Imaging-only model (MAE = 5.76 ± 0.12, R² = 0.46 ± 0.03). **(b)** Model I adjusted for age and sex (MAE = 5.48 ± 0.11, R² = 0.52 ± 0.03). **(c)** Model II further adjusted for all clinical and sociodemographic covariates (MAE = 5.15 ± 0.10, R² = 0.59 ± 0.02). Error bars and shaded regions represent the standard deviates. Model I (adjusted for age, sex). Model II (further adjusted for marital status, stable income, migraine, family history of mental illness, chronic medical history, use of hormone drugs, beta blockers, education level, smoking, alcohol consumption, and coffee consumption).

## 4 Discussion

Our findings establish an independent association between ASCE-detected PFO and anxiety, divergent from traditional risk factors. This challenges the view of PFO as merely a passive embolic conduit and points toward its potential role in anxiety pathophysiology, which may warrant consideration in the clinical assessment of select patients.

The strength of association in our study (adjusted OR = 8.64) substantially exceeds the OR of 4.2 recently reported by Zhai et al. (10) in a smaller cohort (n=89), likely reflecting our larger sample size and more comprehensive confounder adjustment. Unlike their preliminary study, we also examined dose-response across shunt grades and developed an imaging-based predictive model. Similarly, while Sun et al. (9) reported a high prevalence of anxiety in migraine patients with PFO, their study did not isolate the PFO-anxiety link independent of migraine; by adjusting for migraine and other comorbidities, we demonstrate that this association persists beyond migraine, suggesting a direct or independent pathway. Our prospective cohort further extends earlier case-based observations by providing population-level, quantitatively measured evidence, with a 32.0% anxiety prevalence in the ASCE-positive group—comparable to rates in chronic pain populations (approximately 30–40%) and substantially exceeding general population estimates (approximately 10-15%) (8).

The deep learning model trained on ASCE videos achieved clinically meaningful prediction of anxiety scores, despite the absence of overt neurological symptoms in participants. This suggests that PFO-related hemodynamic or microembolic phenomena exert subclinical effects on limbic circuitry—a hypothesis supported by prior reports of anxiety improvement post PFO closure (9). Although the underlying mechanisms remain to be elucidated, the consistently elevated anxiety risk across all positive grades—regardless of shunt burden—supports a potential link between PFO and anxiety that warrants further mechanistic investigation.

Several mechanisms may explain our observations, although these hypotheses are speculative as we did not measure biochemical or neuroimaging biomarkers. One hypothesis is that PFO-related right-to-left shunting might alter serotonin regulation by bypassing pulmonary degradation, potentially affecting limbic circuits (7). Alternatively, shared genetic vulnerabilities (14, 15) or secondary effects of PFO-related conditions (e.g., migraine, sleep disturbances) (8) could mediate the association. These mechanistic proposals require validation in future studies incorporating neuroimaging and biomarker profiling. Our observational design cannot establish causality, and alternative explanations remain equally plausible.

Our study provides several findings that may influence clinical practice: First, the strong independent association between PFO and anxiety (adjusted OR = 8.64) suggests that clinicians should consider routine anxiety screening in patients undergoing ASCE evaluation. Currently, psychiatric assessment is not standard in the workup of PFO patients. Our data indicate that the prevalence of anxiety in ASCE-positive patients (32.0%) is substantially higher than in the general population, supporting the integration of brief anxiety screening tools (e.g., SAS, GAD-7) into the clinical pathway for PFO evaluation. This recommendation is reinforced by the recent findings of Zhai et al. (10), who independently confirmed elevated anxiety in PFO patients, providing external validation for our primary observation. Second, the feasibility of ASCE-based deep learning for anxiety prediction offers a pathway toward automated, imaging-derived psychiatric risk stratification. While not yet ready for clinical deployment without external validation, this approach could eventually enable “opportunistic screening” — deriving anxiety risk information from routine echocardiographic examinations without additional patient burden. Third, the lack of a clear dose-response relationship across shunt grades suggests that the presence of PFO, rather than the magnitude of shunting, may be the primary determinant of anxiety risk. This finding, if confirmed in larger studies, would simplify risk stratification and suggest that all PFO patients merit anxiety screening regardless of shunt grade.

Although all positive ASCE grades were associated with significantly elevated anxiety risk compared with grade 0, we did not observe a clear dose-dependent pattern across grades 1 to 3. This finding should be interpreted with caution, as the subgroup analyses were underpowered due to the modest sample sizes within each grade (Grade 1: n=42, Grade 2: n=24, Grade 3: n=37). Thus, the observed pattern does not support a biological threshold effect but rather reflects statistical uncertainty. Larger cohort studies with adequate power are needed to clarify whether a true non-linear relationship exists between shunt burden and anxiety risk.

There are two main clinical implications. Firstly, ASCE-based anxiety prediction may help identify patients with PFO at high psychiatric risk, enabling targeted interventions. Secondly, these findings suggest that PFO closure trials should incorporate psychiatric endpoints, mirroring the paradigm shift observed in migraine management.

### 4.1 Limitations

Our research has several limitations. First, despite employing 10-fold cross-validation with stable performance (R² SD ≤ 0.03), external validation in larger, multicenter cohorts is essential before clinical translation. Second, subgroup analyses by ASCE grade were underpowered (Grade 1: n=42, Grade 2: n=24, Grade 3: n=37), precluding robust conclusions on dose-response. Third, our frame-sampling approach (step size of 2) may have missed transient hemodynamic signatures. Fourth, the observational design precludes causal inference, and unmeasured confounders may exist. Fifth, single-center design and predominantly Chinese Han population limit generalizability. Sixth, excluding patients with prior psychiatric diagnoses or medications may have introduced selection bias. Seventh, reliance on a single self-report measure (SAS) may be subject to reporting bias and does not capture the full spectrum of anxiety disorders. Eighth, we lacked data on PFO closure and post-closure anxiety outcomes to assess reversibility.

### 4.2 Knowledge gaps

Despite the robust association observed in this study, several knowledge gaps remain: ① The biological pathways linking PFO to anxiety—whether right-to-left shunting affects brain perfusion, microembolic load, or neurotransmitter metabolism—remain unknown, as we did not measure serotonin, inflammatory markers, or neuroimaging correlates. ② Our cross-sectional design cannot determine whether PFO causes anxiety, anxiety causes hyperventilation-induced shunting, or a common factor predisposes to both. ③ Whether higher shunt grades confer greater anxiety risk remains unresolved, pending adequately powered studies. ④ The clinical utility of our deep learning model—its sensitivity, specificity, and predictive values for clinically significant anxiety—has yet to be established. ⑤ Although preliminary reports suggest anxiety improvement post-closure, no controlled prospective study has systematically evaluated psychiatric outcomes after PFO closure. ⑥ The generalizability of our findings to other populations and assessment instruments requires further investigation.

### 4.3 Future directions

Based on these gaps, we propose the following directions: ① Mechanistic studies incorporating neuroimaging (fMRI, transcranial Doppler) and biochemical biomarkers (serotonin, catecholamines, cytokines), alongside pre- and post-closure assessments, to establish causality and reversibility. ② Large-scale multicenter cohorts with adequate power to examine dose-response and validate the deep learning model across diverse populations. ③ Alternative frame-sampling strategies and real-time hemodynamic parameters to capture transient phenomena. ④ Randomized controlled trials of PFO closure with psychiatric endpoints (validated instruments and structured interviews) to determine whether closure modifies anxiety risk. ⑤ Integration of imaging, clinical, and genetic markers into robust prediction models for personalized risk stratification. ⑥ Longitudinal studies to assess temporal relationships, fluctuations in PFO-related anxiety, and the impact of anxiety treatment on clinical outcomes. ⑦ Implementation research on the feasibility, acceptability, and cost-effectiveness of routine anxiety screening and AI-based prediction tools in clinical workflows.

## 5 Conclusion

In summary, this prospective study demonstrates that PFO detected by ASCE is independently associated with anxiety, with ASCE-positive patients showing significantly higher SAS scores and a six-fold higher prevalence of anxiety compared to ASCE-negative patients (32.0% vs. 5.2%, adjusted OR = 8.64). The deep learning model achieved clinically meaningful prediction of SAS scores using ASCE videos (R² = 0.59 ± 0.02 when combined with clinical covariates), suggesting the potential utility of imaging-based psychiatric risk assessment. However, the observational design prevents causal inference, the lack of a clear dose-response relationship requires confirmation in larger cohorts, and external validation of the AI model is essential before clinical translation. These findings establish PFO as a potential risk marker for anxiety and support further research to determine whether PFO modulation can ameliorate anxiety symptoms, but do not yet justify changes to routine clinical practice beyond recommending enhanced awareness and screening in this population. Future research should prioritize mechanistic studies, large-scale validation, and randomized controlled trials with psychiatric endpoints to definitively establish the clinical utility of these findings.

## Data Availability

The raw data supporting the conclusions of this article will be made available by the authors, without undue reservation.
