# Supplementary material for: Association between patent foramen ovale and anxiety: a prospective study with deep learning-assisted analysis of contrast echocardiography
Source: Front Psychiatry. 2026 Sep 30;17:1877855. doi: 10.3389/fpsyt.2026.1877855 (PMC13661545; doi:10.3389/fpsyt.2026.1877855)
Supplement: Supplementary file 1 [file Table1.docx]

**Supplementary Table S1**

| **variable 1** | **variable 2** | **Correlation coefficient (r)** | **p-value** |
| --- | --- | --- | --- |
| Family history ofmental illness | Hormone drugs | 0.43 | 0.00 |
| Family history ofmental illness | PFO | 0.02 | 0.78 |
| Family history ofmental illness | alchol | -0.52 | 0.00 |
| Family history ofmental illness | chronic disease | 0.83 | 0.00 |
| Family history ofmental illness | coffee | -0.38 | 0.00 |
| Family history ofmental illness | receptor blocker | 0.81 | 0.00 |
| Family history ofmental illness | smoking | -0.36 | 0.00 |
| Hormone drugs | PFO | -0.01 | 0.91 |
| Hormone drugs | receptor blocker | 0.52 | 0.00 |
| age | Family history ofmental illness | 0.60 | 0.00 |
| age | Hormone drugs | 0.51 | 0.00 |
| age | PFO | -0.16 | 0.04 |
| age | alchol | -0.48 | 0.00 |
| age | chronic disease | 0.73 | 0.00 |
| age | coffee | -0.65 | 0.00 |
| age | education | -0.84 | 0.00 |
| age | marriage | 0.69 | 0.00 |
| age | migraine | 0.41 | 0.00 |
| age | receptor blocker | 0.75 | 0.00 |
| age | smoking | -0.65 | 0.00 |
| age | stable income | 0.56 | 0.00 |
| alchol | Hormone drugs | -0.41 | 0.00 |
| alchol | PFO | 0.05 | 0.51 |
| alchol | chronic disease | -0.51 | 0.00 |
| alchol | coffee | 0.40 | 0.00 |
| alchol | receptor blocker | -0.53 | 0.00 |
| anxiety | Family history ofmental illness | 0.12 | 0.14 |
| anxiety | Hormone drugs | 0.09 | 0.27 |
| anxiety | PFO | 0.31 | 0.00 |
| anxiety | age | 0.07 | 0.37 |
| anxiety | alchol | 0.00 | 0.97 |
| anxiety | chronic disease | 0.12 | 0.14 |
| anxiety | coffee | -0.01 | 0.91 |
| anxiety | education | -0.05 | 0.52 |
| anxiety | marriage | 0.04 | 0.64 |
| anxiety | migraine | 0.13 | 0.09 |
| anxiety | receptor blocker | 0.12 | 0.14 |
| anxiety | sex | 0.08 | 0.31 |
| anxiety | smoking | -0.06 | 0.49 |
| anxiety | stable income | 0.02 | 0.76 |
| chronic disease | Hormone drugs | 0.49 | 0.00 |
| chronic disease | PFO | -0.09 | 0.27 |
| chronic disease | receptor blocker | 0.97 | 0.00 |
| coffee | Hormone drugs | -0.34 | 0.00 |
| coffee | PFO | 0.09 | 0.28 |
| coffee | chronic disease | -0.41 | 0.00 |
| coffee | receptor blocker | -0.39 | 0.00 |
| education | Family history ofmental illness | -0.65 | 0.00 |
| education | Hormone drugs | -0.46 | 0.00 |
| education | PFO | 0.16 | 0.04 |
| education | alchol | 0.47 | 0.00 |
| education | chronic disease | -0.75 | 0.00 |
| education | coffee | 0.64 | 0.00 |
| education | marriage | -0.57 | 0.00 |
| education | migraine | -0.38 | 0.00 |
| education | receptor blocker | -0.72 | 0.00 |
| education | smoking | 0.60 | 0.00 |
| education | stable income | -0.49 | 0.00 |
| marriage | Family history ofmental illness | 0.32 | 0.00 |
| marriage | Hormone drugs | 0.26 | 0.00 |
| marriage | PFO | -0.15 | 0.05 |
| marriage | alchol | -0.13 | 0.11 |
| marriage | chronic disease | 0.39 | 0.00 |
| marriage | coffee | -0.56 | 0.00 |
| marriage | migraine | 0.29 | 0.00 |
| marriage | receptor blocker | 0.39 | 0.00 |
| marriage | smoking | -0.41 | 0.00 |
| marriage | stable income | 0.76 | 0.00 |
| migraine | Family history ofmental illness | 0.42 | 0.00 |
| migraine | Hormone drugs | 0.67 | 0.00 |
| migraine | PFO | 0.10 | 0.20 |
| migraine | alchol | -0.38 | 0.00 |
| migraine | chronic disease | 0.51 | 0.00 |
| migraine | coffee | -0.25 | 0.00 |
| migraine | receptor blocker | 0.54 | 0.00 |
| migraine | smoking | -0.30 | 0.00 |
| receptor blocker | PFO | -0.09 | 0.27 |
| sex | Family history ofmental illness | -0.11 | 0.17 |
| sex | Hormone drugs | 0.34 | 0.00 |
| sex | PFO | 0.07 | 0.36 |
| sex | age | -0.07 | 0.40 |
| sex | alchol | -0.41 | 0.00 |
| sex | chronic disease | -0.08 | 0.30 |
| sex | coffee | -0.07 | 0.41 |
| sex | education | 0.04 | 0.58 |
| sex | marriage | -0.08 | 0.31 |
| sex | migraine | 0.33 | 0.00 |
| sex | receptor blocker | -0.08 | 0.30 |
| sex | smoking | -0.29 | 0.00 |
| sex | stable income | -0.10 | 0.21 |
| smoking | Hormone drugs | -0.37 | 0.00 |
| smoking | PFO | 0.16 | 0.05 |
| smoking | alchol | 0.58 | 0.00 |
| smoking | chronic disease | -0.48 | 0.00 |
| smoking | coffee | 0.68 | 0.00 |
| smoking | receptor blocker | -0.46 | 0.00 |
| stable income | Family history ofmental illness | 0.20 | 0.01 |
| stable income | Hormone drugs | 0.20 | 0.01 |
| stable income | PFO | -0.11 | 0.18 |
| stable income | alchol | -0.03 | 0.70 |
| stable income | chronic disease | 0.27 | 0.00 |
| stable income | coffee | -0.48 | 0.00 |
| stable income | migraine | 0.19 | 0.01 |
| stable income | receptor blocker | 0.27 | 0.00 |
| stable income | smoking | -0.37 | 0.00 |
